# Baseline health parameters of rhinoceros auklets (*Cerorhinca monocerata*) using serum protein electrophoresis, acute phase proteins, and biochemistry

**DOI:** 10.3389/fvets.2024.1379980

**Published:** 2024-06-25

**Authors:** Lisa K. F. Lee, J. Mark Hipfner, Greg Frankfurter, Carolyn Cray, Scott F. Pearson, Christine Fiorello, Nikolas M. T. Clyde, Sarah A. Hudson, Sarah E. Parker, David E. Stallknecht, Emmanuelle Furst, Katherine H. Haman

**Affiliations:** ^1^Department of Veterinary Pathology, Western College of Veterinary Medicine, University of Saskatchewan, Saskatoon, SK, Canada; ^2^Wildlife Research Division, Environment and Climate Change Canada, Delta, BC, Canada; ^3^School of Veterinary Medicine, Karen C. Drayer Wildlife Health Center, Davis, CA, United States; ^4^Division of Comparative Pathology, Department of Pathology and Laboratory Medicine, Miller School of Medicine, Miami, FL, United States; ^5^Wildlife Program, Science Division, Washington Department of Fish and Wildlife, Olympia, WA, United States; ^6^Hawks Aloft, Inc., Albuquerque, NM, United States; ^7^Centre for Applied Epidemiology, Large Animal Clinical Sciences, Western College of Veterinary Medicine, University of Saskatchewan, Saskatoon, SK, Canada; ^8^Department of Population Health, Southeastern Cooperative Wildlife Disease Study, College of Veterinary Medicine, University of Georgia, Athens, GA, United States; ^9^Alaska SeaLife Center, Seward, AK, United States

**Keywords:** acute phase proteins, baseline, *Cerorhinca monocerata*, protein electrophoresis, rhinoceros auklets, serum biochemistry

## Abstract

Clinical metrics of baseline health in sentinel seabird species can offer insight into marine ecosystem dynamics, individual and population health, and assist in wildlife rehabilitation and conservation efforts. Protein electrophoresis is useful for detecting changes in acute phase proteins and immunoglobulin levels that may indicate subtle inflammatory responses and/or infectious disease. Serum biochemistry can highlight nutritional status, metabolic derangements, and organ injury and function. However, baseline values for such health parameters are largely unknown for many seabird species. Therefore, the objective of this study is to establish baseline clinical health reference intervals for serum protein electrophoresis, acute phase proteins including serum amyloid A and haptoglobin, and biochemistry parameters in the rhinoceros auklet (*Cerorhinca monocerata*), a key sentinel species in the North Pacific. From 2013 to 2019, 178 wild, apparently healthy breeding adult rhinoceros auklets were captured across four breeding colonies in British Columbia, Canada (Lucy Island, Pine Island, Triangle Islands, and SGang Gwaay) and from one colony in Washington, United States (Protection Island). Reference intervals were calculated for protein electrophoresis fractions and acute phase proteins (*n* = 163), and serum biochemistry (*n* = 35) following established guidelines by the American Society of Veterinary Clinical Pathology. Animals were also assessed for the presence of antibodies to the influenza A virus. Approximately 48% (70/147) of sampled birds were seropositive for influenza A virus, with a prevalence of 50% (6/12) in 2013, 75% (47/63) in 2014, and 24% (17/72) in 2019. This work provides clinical baseline health metrics of a key North Pacific sentinel species to help inform marine ecosystem monitoring, recovery, and rehabilitation efforts in the Pacific Northwest.

## 1 Introduction

Serum baseline values can illustrate key features about wild seabird health at the individual and population levels. Serum biochemistry provides information about electrolyte balance, metabolism, and internal organ status (1). Acute phase proteins (APP) and protein electrophoresis (EPH) can be used to gauge inflammatory response and prognosis in avian species, and are becoming more widely used (2–4). Protein electrophoresis and APP allow researchers and clinicians to detect inflammatory changes earlier when compared to traditional hematology and serum biochemistry approaches (5). This is due to the rapid changes that occur in APP during acute disease and inflammation, such as the upregulation of serum amyloid A (SAA), haptoglobin (HP) (PIT54 is the homologous protein in birds) (6), and α- and β-globulins; and downregulation of albumin (7). Particularly in marine environments, APP such as HP provide valuable information in population health assessments of marine mammal species exposed to oil and other pollutants (8).

Maintaining established clinical baseline health parameters may serve as a pre-defined recovery goal for seabirds in the event of a marine environmental disruption (9–11). For example, oil contamination from both acute and chronic releases has long-term physiological and metabolic impacts on the health, reproduction, and survival of migratory seabirds (12–17). Baseline clinical health parameters of free-ranging seabirds can help distinguish between local or large-scale environmental impacts to marine systems, such as point-source anthropogenic stressors including oil spills and other marine pollutants (18–20). Furthermore, this information may facilitate wildlife rehabilitation and recovery efforts (21, 22). This becomes especially critical during environmental disasters such as oil spills, as it provides baseline metrics for recovery.

Climate change and severe weather are other significant contributors to global seabird species declines (23) due to decreased habitat suitability, prey abundance, and shifts in pathogen-host dynamics associated with ocean warming (24, 25). Seabirds may act as ecosystem sentinels for their pelagic and coastal habitats due to their responsiveness to environmental changes and their role as top predators in marine food webs (26). This is reflected through population health and ultimately, changes in breeding success and survival (27–29).

Seabirds can also potentially aid in disease surveillance across all flyways. Within the Pacific Flyway, influenza A virus (IAV) is of particular interest. While wild aquatic birds can act as disease reservoirs in the Pacific Flyway (30, 31), knowledge about the role of different seabird species in IAV ecology remains limited due to difficulties in sampling free-ranging seabirds (32, 33). Seabirds such as gulls are known to act as reservoirs for low pathogenic avian influenza H13 and H16 subtypes, and were able to spread highly pathogenic avian influenza (HPAI) H5 subtype rapidly due to large distances traveled (34). The ongoing H5N1 2.3.4.4b HPAI outbreak across North America further highlights the importance of IAV in seabirds (35), especially since there is a potential for catastrophic loss of species infected with H5N1 HPAI (36–38) (e.g., brown pelicans, Caspian terns). However, few studies have investigated baseline exposure levels to IAV in general.

In the North Pacific, the rhinoceros auklet (*Cerorhinca monocerata*) from the Alcidae family is an important indicator species for ecosystem health (18, 39). Ocean warming can lead to long-term decreases in rhinoceros auklet abundance (40), as they are sensitive to oceanographic changes that shift trophic level interactions and diet composition. This can also affect reproductive success, as reproductive performance is correlated with diet quality and availability (41–43). Interestingly, adult survival rates remained relatively stable during extreme environmental variation (44). Alcids have been particularly vulnerable to large mortality events in recent years (45) with disease playing a major role in a rhinoceros auklet mortality event in the Salish Sea (39). Assessment of clinical parameters in rhinoceros auklets can potentially provide a more comprehensive view of marine ecosystem health, especially when integrated with other attributes such as demography, reproduction, and morphometrics.

Few studies provide baseline serum biochemistry, EPH, and APP parameters for seabirds due to challenges in obtaining samples and limited APP reagent validation in avian species (46). There is also a scarcity of clinical baseline seabird population health data based on the American Society of Veterinary Clinical Pathology guidelines in reference interval (RI) generation (47), which are founded on the use of significant sample sizes and recommended statistical methods. The objectives of this study were therefore to: (1) establish baseline clinical health serum biochemistry, EPH, and APP RIs for the rhinoceros auklet on multiple breeding colonies in the core of its breeding range; and (2) assess IAV antibody prevalence among these breeding colonies.

## 2 Materials and methods

### 2.1 Animal capture and blood collection at breeding colonies

Research protocols employed in this study were approved by Simon Fraser University Animal Care Services (#974B-94), the Western and Northern Animal Care Committee of Environment and Climate Change Canada’s Canadian Wildlife Service (14MH01, and 19MH01), ECCC Migratory Birds banding permit (10667F), and US Fish and Wildlife Federal Bird Banding Permit (22913).

**Figure 1:**
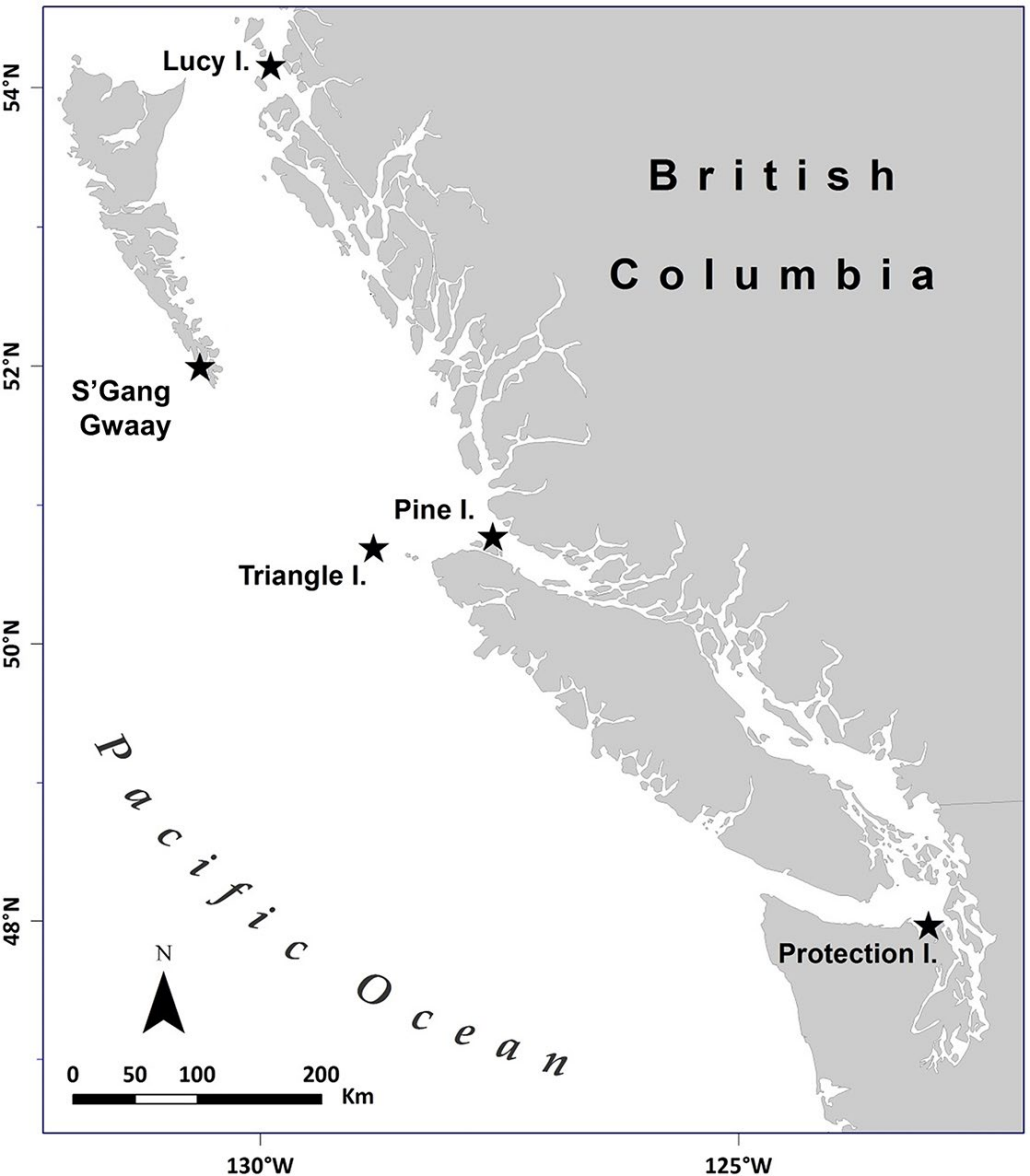
Map of rhinoceros auklet (*Cerorhinca monocerata*) breeding colonies studied in British Columbia, Canada, and Washington, United States within the North Pacific from 2013 to 2019. The seabird breeding colonies were located on Lucy Island (54.294418°N, −130.621907°W), Pine Island (50.976062°N, −127.729909°W), Triangle Island (50.851023°N, −129.066292°W), SGang Gwaay (52.092634°N, −131.225633°W) in British Columbia, and Protection Island (48.126341°N, −122.930289°W) in Washington, United States. Adapted from Environmental Pollution, Volume 239, Hipfner et al., “Two forage fishes as potential conduits for the vertical transfer of microfibres in Northeastern Pacific Ocean food webs”, Pages 215-222, Copyright Elsevier (2018).

Adult rhinoceros auklets were caught on land at breeding colonies at night, either by hand, with landing nets, or mist nets in July (2013, 2014, and 2019) across four colonies in British Columbia, Canada (Lucy, Pine, and Triangle Islands, plus SGang Gwaay) and one colony in Washington, United States (2019, Protection Island) in the North Pacific (Figure 1). Birds were weighed with a 1 kg (± 5 g) analog spring scale (Pesola AG, Switzerland), and the following morphometric measurements were collected: maximum flattened wing chord, tarsus, culmen length, bill depth at gonys, and horn height. Blood was taken from the brachial vein, using 27 g needles and 3 cc syringes. Whole blood was centrifuged at 10,000 rotations per minute for 5–10 min to separate erythrocytes from serum, within 4–6 h of collection. Serum was collected into tubes after the blood samples clotted, then subsequently frozen in liquid nitrogen dry shippers. Samples were stored at −20°C until shipping to the University of Miami Avian and Medicine Laboratory (Miami, FL, United States), California Animal Health and Food Safety Laboratory (Davis, CA, United States), University of Georgia (Athens, GA, United States), and/or the University of Lethbridge (Lethbridge, AB, United States). Total elapsed time between sample collection and analysis from wild birds ranged from 160 to 598 days (median: 173 days).

### 2.2 Preliminary serum amyloid A and haptoglobin evaluation in captive rhinoceros auklets

Five captive rhinoceros auklets at the Alaska SeaLife Center were selected for measurement of SAA and HP levels from May 2007 to September 2013, due to the presence of various clinical abnormalities associated with inflammation, in most cases involving their feet (Supplementary Tables S2–S4). Four of the five birds were originally collected as eggs from Middleton Island, AK in June of 2006 and participated in a nutritional research project prior to being placed at the Alaska SeaLife Center for long-term care in early 2007. The fifth bird selected was hatched at the Alaska SeaLife Center from a mated pair of the previously described individuals. One plasma or serum sample was collected from each bird during clinical disease and when clinically normal. Serum or plasma was collected from peripheral veins (brachial, metatarsal, or jugular vein) and processed as described previously.

### 2.3 Laboratory sample processing

**Figure 2:**
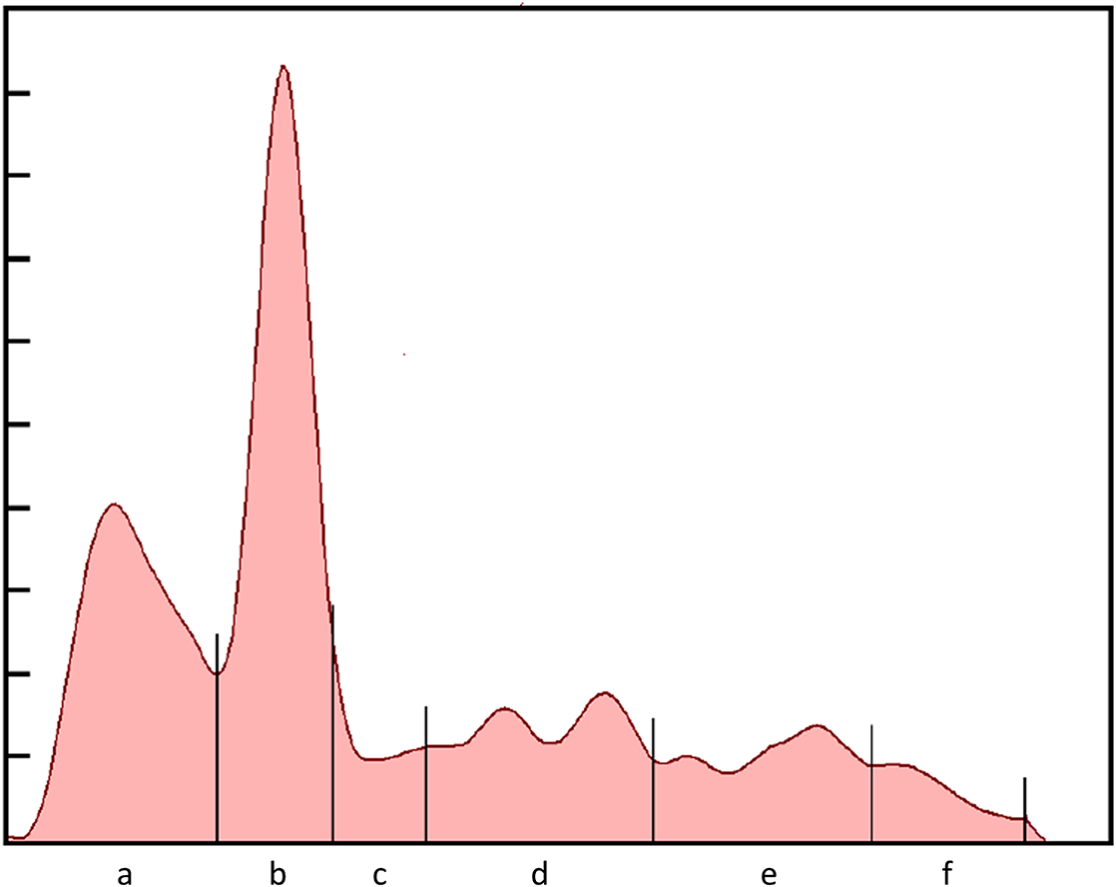
Electrophoretogram of a free-ranging adult rhinoceros auklet (*Cerorhinca monocerata*) that was presumed healthy. Protein fractions are: **(A)** prealbumin; **(B)** albumin; **(C)** α-1; **(D)** α-2; **(E)** β-; and **(F)** γ-globulins.

Serum biochemistry, protein electrophoresis, and acute phase protein analysis was conducted at the University of Miami—Avian and Medicine Laboratory (Miami, FL). Routine biochemistry testing was performed using a Vitros 250 analyzer (Ortho, Rochester, NY). Protein electrophoresis was conducted using the SPIFE 3000 system and split beta gels (Helena Laboratories, Beaumont, TX, United States). A representative electrophoretogram from a rhinoceros auklet is presented in Figure 2. Fraction delimits were placed according to conventions established for other avian species (2) and included prealbumin, albumin, and α-1, α-2, β-, and γ-globulins. The albumin to globulin ratio was calculated as the sum of prealbumin and albumin divided by the sum of the globulins. The absolute values for the fractions were calculated by multiplying the fraction percent values by the total protein. Haptoglobin levels were determined using the phase colorimetric assay (Tridelta, Morris Plains, NJ), and SAA levels were determined using the SAA-LZ immunoturbidimetric assay (Eiken Chemical Co, Tokyo, Japan). Both assays were performed on a Daytona Rx analyzer (Kearneysville, WV). Assay reactivity, as determined by stepwise dilution of high abnormal samples (100, 90, 80, …) was found to be linear under dilution. For SAA, the slope included 1 (0.89–1.21) and the *y*-intercept included 0 (−9.81–43.99). The runs test did indicate a significant deviation from linearity (*p* = 0.02). For HP, the slope included 1 (0.48–1.11) and the *y*-intercept included 0 (−0.11–0.62). The runs test did not indicate a significant deviation from linearity (*p* = 0.90). Coefficient of variation and diagnostic limits were consistent with that observed with other species (3); analysis was performed with GraphPad Prism 8.0 (GraphPad Software, San Diego, California, United States).

Sera were tested for antibodies to the IAV nucleoprotein using a commercial bELISA (IDEXX AI MultiS-Screen Ab test, IDEXX Laboratories, Westbrook, Maine, United States) according to the manufacturer’s instructions either at the California Animal Health and Food Safety Laboratory (Davis, CA) or the University of Georgia (Athens, GA). Sera were considered positive for antibodies to IAV if the serum-sample-to-negative-control (S/N) absorbance value was <0.7, based on recommendations for evaluating wildlife species (48).

DNA was extracted from blood stored in Queen’s lysis buffer using a modified Chelex protocol (49, 50) at the University of Lethbridge (Lethbridge, AB). Individuals were sexed using the Z43BF/Z43BR Primer Pair (51); the forward primer modified with M13 to allow incorporation of fluorescent marker to run on Licor gel. All PCR reactions were conducted in 10 μL reactions with 1 μL of genomic DNA. PCR cocktails contained 2.0 μL ClearFlexi Buffer 5x (Promega), 2.5 mM MgCl₂, 200 μM dNTP, 1 μM each primer, 0.05 μM M13 primer, and 0.5 units GoTaq (Promega). The following Thermocycler Conditions were used: 1 cycle of 30 s at 94°C; 35 cycles of 30 s at 94°C, and 45 s at 55°C, and 45 s at 72°C, with a final extension for 5 min at 72°C, and 5 s at 4°C. All PCR products were run on a 6% acrylamide gel. Two positive controls (one male and one female) and a negative control were included on each run.

### 2.4 Statistical analyses

Statistical analyses were conducted using RStudio (R Development Core Team, Vienna, Austria) and Stata (17.0, StataCorp LLC, College Station, TX, United States), with results deemed significant at *p* ≤ 0.05, unless otherwise stated. Sex was predicted based on the following morphometric measurements (52): A designation of female was assigned if bill depth was <16.5 mm, while males had a bill depth of >17.0 mm. Birds with ambiguous bill depths (i.e., 16.5–17.0 mm) were assigned female if they weighed <500 g, and assigned male if ≥500 g. The McNemar’s test was conducted to determine if there was a difference between genetic sex of rhinoceros auklets and when predicted by morphometric measurements.

Normality was first assessed for serum EPH, APP, and biochemistry measurands using the Shapiro–Wilk test. Measurands were considered to have a non-Gaussian distribution if *p* ≤ 0.3, as per guidelines from the American Society of Veterinary Clinical Pathology (53). As the majority of the parameters had a non-Gaussian distribution, the Kruskal-Wallis test was used to determine differences between colonies, years sampled, and morphometrically predicted sex. Pairwise comparisons were evaluated with Dunn’s test, with *p*-values adjusted with the Benjamini-Hochberg method to control the type I error rate. The distribution of IAV seropositivity status was analyzed using a multilevel mixed-effects logistic regression with colony, year, and morphometrically predicted sex, when clustering by colony. Variables were considered for multivariable model building if association with IAV seropositivity was at a threshold of *p* ≤ 0.20. Model fit was evaluated with Aikake’s information criteria (AIC). Inclusion of variables was tested with a likelihood ratio for successive models with forward stepwise selection.

Data collected from individuals of all colonies were pooled together to generate RIs for serum biochemistry, EPH, and APP. Reference intervals containing the central 95% of the population were calculated using Reference Value Advisor v. 2.1 Microsoft Excel add-on (54), according to guidelines established by the American Society of Veterinary Clinical Pathology (47). A nonparametric method was used as most distributions were non-normal. Lower and upper bounds represent the 2.5 and 97.5th percentiles, respectively. Outliers were identified using Tukey’s interquartile fences and excluded from RI calculations following histogram and boxplot examination. For variables with *n* ≥ 120, a nonparametric method was used for generating the 95% RI, with 90% confidence intervals (CI) of the upper and lower limits of the RI. For non-normally distributed variables with *n* ≥ 20 and < 40, the robust method was used to calculate RI and 90% CI upper and lower limits of the RI (47).

## 3 Results

**Figure 3:**
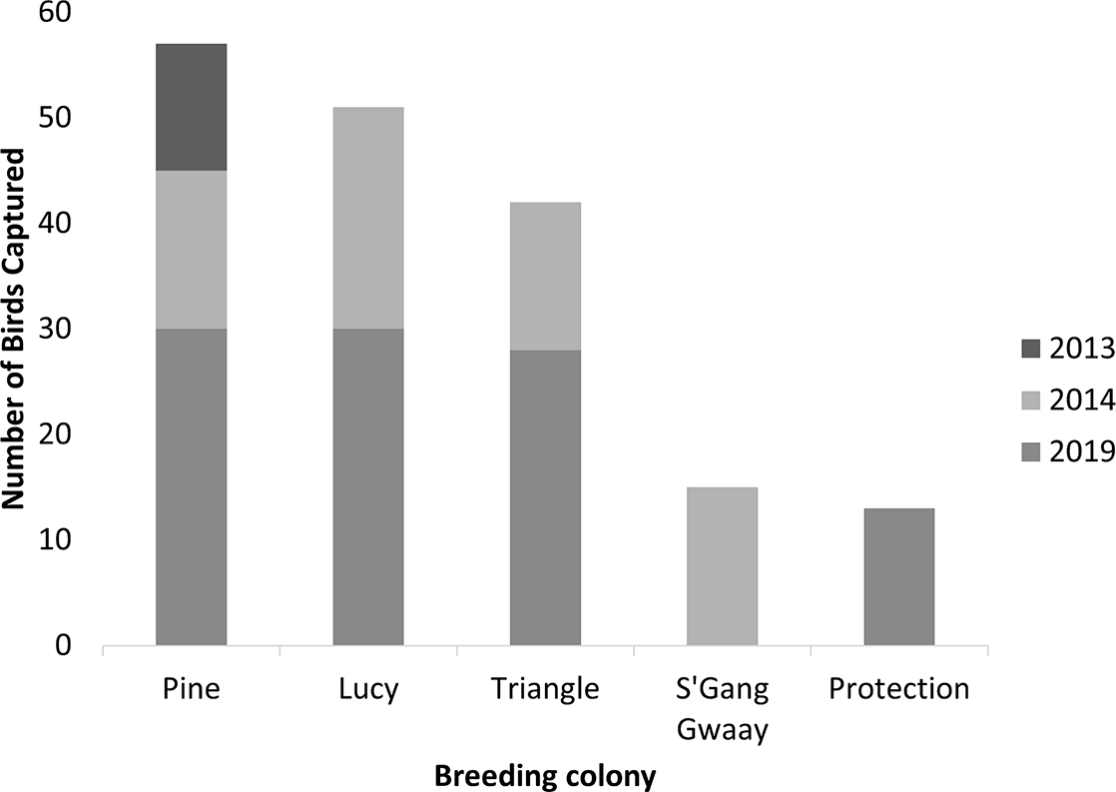
Number of wild breeding rhinoceros auklets (*Cerorhinca monocerata*) captured by year and colony from Lucy, Pine, Triangle Islands, and SGang Gwaay, British Columbia, Canada and from Protection Island, Washington, United States, 2013–2019.

From 2013 to 2019, 178 adult rhinoceros auklets were captured from breeding colonies at the sampling sites (Figures 1, 3). On brief physical exam, no gross external abnormalities were noted and all individuals were presumed healthy. All animals had evidence of breeding present (i.e., presence of bill load for chick feeding) at time of capture. Descriptive statistics for mass and morphometric values are summarized in Table 1.

**Table 1:** Descriptive statistics for morphometric data of wild breeding adult rhinoceros auklets (*Cerorhinca monocerata*) from Lucy, Pine, Triangle, and SGang Gwaay Islands in British Columbia and Protection Island, Washington, 2013–2019.

	*n*	Mean	Median	Standard deviation	Min	Max
Mass (g)	178	502	500	32	405	570
Wing Cord (mm)	177	185	185	5	167	199
Tarsus (mm)	157	31.6	31.4	1.8	27.7	40.1
Culmen (mm)	168	32.1	32.6	3.0	20.5	36.1
Horn (mm)	168	26.5	26.6	2.1	20.8	32.6
Bill Depth (mm)	168	16.5	16.5	1.2	13.2	20.7

Of the captured birds, the sex of 38 animals was determined genetically, and was composed of 19 males (50%) and 19 females (50%). Bill depth was not recorded for 2/38 of the genetically sexed birds. Based on bill depth and weight measurements as described above, there were 93 females (52%), and 75 males (42%). Ten animals (6%) were of unknown sex, as morphometric data for these birds were not collected. Supplementary Table S1 shows the cross classification of genetic sex compared to morphometric predictions in 36 animals. The McNemar’s test indicated that the proportion determined as male and female by the two tests was not significantly different (*p* = 1). When comparing genetic versus morphometrically predicted sex, there was a weak level of agreement (kappa = 0.44).

Blood samples were collected from 163 individuals. Lipemia occurred in two samples with a lipemic index of 1 and 2, respectively (0–4 scale, with 4 graded as severe); these animals had total protein levels comparable to other animals in the study. Most serum samples had no evidence of hemolysis; however, four and five birds had hemolysis indexes of 1+ (mild) and 2+ (moderate), respectively. Serum protein electrophoresis and biochemistry values were assessed in 163 and 35 animals, respectively. Distributions of values were non-normal, so non-parametric techniques were used. Reference intervals containing the central 95% of the population, with corresponding 90% CI for the lower and upper limit of the RI, are presented for protein electrophoresis fractions and APP (Table 2) and biochemistry data (Table 3). For individual analyte values below the limit of detection, the RI was calculated using the limit of detection (e.g., <0.1 mg/L SAA was entered as 0.1 mg/L for RI calculation). Histograms showing the distribution of serum biochemistry values are provided in Supplementary Figures S1, S2 due to smaller sample sizes (*n* < 40) assessed. Gamma-glutamyl transferase was assayed but RI were not calculated as 80% (28/35) of samples were below the limit of detection (< 5 U/L). The remaining gamma-glutamyl values ranged from 5 to 9 U/L. For creatinine, 13/35 (37%) samples were below the limit of detection (< 0.2 mg/dL), so RI for the blood urea nitrogen to creatinine ratio was not calculated. Serum concentrations for SAA and HP for all birds assessed are shown in Supplementary Figure S3.

**Table 2:** Serum protein electrophoresis fractions and acute phase protein reference intervals (RI) for wild breeding adult rhinoceros auklets (*Cerorhinca monocerata*) from Lucy, Pine, Triangle, and SGang Gwaay Islands in British Columbia and Protection Island, Washington, 2013–2019.

Measurand	Units	*n*	Mean	SD	Median	Min	Max	*p*-value^a^	Distribution^a^	Method^b^	LRL of RI^b^	URL of RI^b^	CI 90% of LRL^b^	CI 90% of URL^b^
Total protein	g/dL	161	4.48	1.38	4	1.4	9.8	<0.001	NG	NP	2.4	7.4	1.4–2.6	7.0–9.8
Albumin to globulin ratio		163	1.2	0.33	1.2	0.45	1.98	0.29	NG	NP	0.6	1.8	0.5–0.7	1.7–2.0
Prealbumin	%	159	7.6	4.3	6.8	0.3	22.4	<0.001	NG	NP	0.4	18.3	0.3–0.5	16.2–22.4
	g/dL	159	0.34	0.22	0.29	0.01	1.1	<0.001	NG	NP	0.02	0.9	0.01–0.02	0.82–1.10
Albumin	%	163	44	7.1	45.1	25.4	59.1	0.004	NG	NP	28.5	55.9	25.4–31.5	53.7–59.1
	g/dL	162	2	0.6	1.9	0.6	4.2	<0.001	NG	NP	0.8	3.7	0.6–1.1	3.3–4.2
α-1 globulins	%	161	6.5	1.9	6.2	3	12.7	<0.001	NG	NP	3.4	11.9	3.0–4.0	10.7–12.7
	g/dL	162	0.3	0.14	0.26	0.07	0.89	<0.001	NG	NP	0.1	0.7	0.07–0.15	0.56–0.89
α-2 globulins	%	157	15.6	2.9	15.2	11.1	25.9	<0.001	NG	NP	11.5	24.9	11.1–12.3	23.4–25.9
	g/dL	161	0.74	0.3	0.64	0.21	1.95	<0.001	NG	NP	0.35	1.6	0.21–0.40	1.30–1.95
β-globulins	%	163	15.8	4.1	15	8.7	30.6	<0.001	NG	NP	9.7	26.6	8.7–10.2	23.6–30.6
	g/dL	157	0.69	0.27	0.64	0.23	1.73	<0.001	NG	NP	0.3	1.5	0.23–0.34	1.11–1.73
γ-globulins	%	163	7.9	2.2	7.8	3.1	14.9	0.08	NG	NP	3.8	13.3	3.1–4.4	12.0–14.9
	g/dL	161	0.36	0.16	0.34	0.1	0.99	<0.001	NG	NP	0.1	0.8	0.10–0.16	0.67–0.99
Serum amyloid A (SAA)	mg/L	143	1.24	1.99	0.1	<0.1	10.08	<0.001	NG	NP	<0.1	6.7	0.1–0.1	5.9–10.1
Haptoglobin (HP)	mg/mL	141	0.07	0.15	0.01	<0.01	0.27	<0.001	NG	NP	<0.01	0.23	0.01–0.01	0.19–0.27

^a^The Shapiro–Wilk test was used to assess normality of the distributions at a threshold of *p* < 0.3. NG, Non-Gaussian. ^b^Outliers were identified by Tukey interquartile fences and removed. The nonparametric (NP) method was used to generate reference intervals (RI), containing the central 95%. LRL, Lower reference limit; URL, Upper reference limit; CI, Confidence interval; SD, Standard deviation.

**Table 3:** Serum biochemistry reference intervals (RI) of wild breeding adult rhinoceros auklets (*Cerorhinca monocerata*) from Lucy, Pine, Triangle Islands, and SGang Gwaay in British Columbia, 2013–2019.

Measurand	Units	*n*	Mean	SD	Median	Min	Max	*p*-value^a^	Distribution^a^	Method^b^	LRL of RI^b^	URL of RI^b^	CI 90% of LRL^b^	CI 90% of URL^b^
Sodium	mmol/L	35	137.5	3.2	137	133	145	0.08	NG	R	130.4	143.6	128.6–131.8	141.8–145.6
Potassium	mmol/L	35	3.03	0.47	2.9	2.2	4.1	0.31	G	R	1.98	3.99	1.76–2.22	3.67–4.23
Chloride	mmol/L	35	117.7	3.2	117	113	126	0.02	NG	R	110.5	123.9	109.0–112.4	122.1–125.8
CO_2_	mmol/L	35	19.6	5.1	20	<5	29	0.02	NG	R	9.8	31	7.1–13.0	27.7–33.6
Calcium	mg/dL	34	9.38	0.84	9.4	7.1	10.7	0.001	NG	R	7.76	11.21	7.31–8.24	10.68–11.64
Phosphorus	mg/dL	33	3.02	1.58	3	0.8	7.3	<0.001	NG	R	0	5.87	0–0.49	4.89–6.84
Magnesium	mg/dL	35	2.01	0.31	2	1.3	2.7	0.99	G	R	1.36	2.65	1.23–1.57	2.50–2.81
Uric acid	mg/dL	34	15.06	7.77	14.6	1.2	37.9	<0.001	NG	R	0	30.1	0–2.94	25.38–34.98
Urea nitrogen	mg/dL	34	5.3	2.4	5	1	12	0.002	NG	R	0	9.5	0–1.3	8.1–11.4
Creatinine	mg/dL	32	0.25	0.08	0.2	<0.2	0.4	<0.001	NG	R	n/a	n/a	n/a	n/a
AST	U/L	35	349.2	239.8	296	49	1,275	<0.001	NG	R	0	804.4	n/a	609.1–1005.7
CK	U/L	33	253.7	159.4	208	39	752	<0.001	NG	R	0	539.7	n/a	416.9–665.6
Glucose	mg/dL	32	383.7	62.9	399	231	488	<0.001	NG	R	256.2	525	222.4–297.5	490.3–556.6
Amylase	U/L	35	2082	805	2,286	856	3,519	0.005	NG	R	410.4	3847.5	48.2–987.4	3575.7–4181.3
Lipase	U/L	33	11.5	10.6	8	<1	41	<0.001	NG	R	0	29.9	n/a	22.9–38.5
Triglyceride	mg/dL	33	171.6	59.3	163	72	371	<0.001	NG	R	35.1	280.8	0–83.3	230.8–323.5
Cholesterol	mg/dL	35	303.4	58.3	305	184	440	0.8	G	R	183.6	424.7	158.7–213.8	396.6–453.4

^a^The Shapiro–Wilk test was used to assess normality of the distributions at a threshold of *p* < 0.3. NG, Non-Gaussian; G, Gaussian. ^b^Outliers were identified by Tukey interquartile fences and removed. The robust (R) method was used to generate reference intervals (RI), containing the central 95%. LRL, Lower reference limit; URL, Upper reference limit; CI, Confidence interval; SD, Standard deviation; AST, Aspartate aminotransferase; and CK, Creatine kinase.

For comparison to birds with known illnesses, SAA and HP levels from five captive rhinoceros auklets at the Alaska SeaLife Center were assessed during clinical disease and when clinically healthy (Supplementary Tables S2–S4). Four of the five clinically ill rhinoceros auklets were diagnosed with pododermatitis with active abscessation, necrosis, cellulitis, or an active joint infection; one clinically ill rhinoceros auklet had an open diagnosis with nonspecific signs of illness that responded to supportive therapy. SAA ranged from 0.8 to 385.1 mg/L (median 43.9 mg/L) and HP ranged from 0.35 to 2.45 mg/mL (median 1.39 mg/mL). When considered clinically normal, SAA ranged from <0.1 to 15.52 mg/L (median 1.65 mg/L) and HP ranged from 0.35 to 0.87 mg/mL (median 0.5 mg/mL). EPH was also performed in three of the clinically ill birds (Supplementary Table S3).

Seropositivity for IAV was determined by enzyme-linked immunosorbent assay in 147 individuals. Year, colony, and morphometrically predicted sex were assessed for association with IAV seropositivity. Sex was not associated (*p* = 0.74). Year and colony were considered for further multivariate model building as *p* values were below a threshold of *p* ≤ 0.2. After adjusting for colony clustering, the prevalence of IAV in 2014 (75%; 47/63) was significantly higher than in 2019 (24%; 17/72; *p* < 0.001), but not different compared to in 2013 (50%; 6/12; *p* = 0.1). A subset analysis including only colonies with data for multiple years and only the last 2 years of sampling did not change estimates of effect (<5% difference; data not shown). The analysis of the full dataset is presented here. The model including year of sampling yielded a better fit than the base model (AIC = 173 vs. AIC = 205). Overall, including the year of sampling yielded a significantly better model (Likelihood ratio test, *p* < 0.0001).

## 4 Discussion

This study provides protein electrophoresis, APP, and serum biochemistry RIs and IAV seroprevalence among wild adult rhinoceros auklets from large breeding colonies in the North Pacific. While various studies have described protein electrophoresis fractions in avian species previously, sample sizes have generally been limited and reference ranges only compiled for a handful of species including Xantus’s murrelets (*Synthliboramphus hypoleucus*), common loons (*Gavia immer*), and captive American flamingos (*Phoenicopterus ruber*) (3, 4, 55). This study, to our knowledge, provides one of the largest samplings of a free-ranging seabird to assess baseline health by protein electrophoresis to date. It is important to note though, that reference intervals are both species and laboratory-specific, and that RI calculation method (e.g., whether outliers were removed) likely affects calculated intervals. We therefore followed current guidelines from the American Society of Veterinary Clinical Pathology for RI generation.

Analysis of acute phase response is a more recent advancement in avian medicine to complement disease diagnosis, such as in the diagnosis of aspergillosis or chlamydiosis (2, 5). Pododermatitis is negatively associated with albumin levels in captive American flamingos (3), which has potential implications for wildlife rehabilitation as this disease is a common negative consequence of captivity in waterbirds (56). Serum EPH and APP profiles of the rhinoceros auklets in this study were unremarkable when compared to other species, with some exceptions. Albumin concentrations and the associated albumin to globulin ratio range were slightly elevated in rhinoceros auklets compared to wild adult Xantus’s murrelets, common loons, brown pelicans (*Pelecanus occidentalis*), juvenile herring gulls (*Larus argentatus*), and Caspian terns (*Sterna caspia*) (4, 55, 57, 58). By contrast, captive American flamingos exhibited a higher albumin to globulin ratio, in addition to prealbumin and HP concentrations (3).

Serum amyloid A (SAA) and HP are other biomarkers for monitoring inflammation in birds (59). Inflammation has been linked with elevations of SAA levels in peregrine falcons (*Falco peregrinus*) with fungal pneumonia and pododermatitis (46). Rhinoceros auklets in this study had a comparable SAA RI to healthy peregrine falcon individuals, although some rhinoceros auklets had highly elevated SAA concentrations (> 20 mg/L) subsequently identified as statistical outliers. This could represent inflammatory disease, and potentially subclinical illness. As birds were presumed healthy based on brief external examination and breeding status, this represents a limitation within the baseline health parameters generated in this study.

Haptoglobin values in this study were often below the limit of detection of the analyzer. In general, HP is considered a minor acute phase protein; HP binds free hemoglobin, minimizing oxidative damage caused during inflammation (6, 60). Plasma HP concentration has been previously associated with herpesvirus infection in frigatebird nestlings and was predictive of short-term survival (61). Haptoglobin concentrations also increased in adult mallard ducks (*Anas platyrhyncos*) experimentally injected with bacterial lipopolysaccharide (62). By contrast, these mallard ducks exhibited no change in HP when experimentally exposed to fuel oil (62). Wild common guillemots (*Uria aalge*) had plasma HP concentrations negatively correlated with exposure to polycyclic aromatic hydrocarbons in crude oil, thought to be related to Heinz body hemolytic anemia (63). Further investigation is required to understand the contextual implications of HP concentration alterations in seabirds, particularly related to oiling and infection by different pathogens.

Since there is limited information about SAA and HP values in clinically normal compared to abnormal seabirds, serum or plasma concentrations were assessed for five captive rhinoceros auklets. For SAA, all individuals with a definitive diagnosis of an acute infection had much greater SAA levels compared to the RI calculated in this study. One clinically abnormal bird with nonspecific clinical signs of illness and no definitive diagnosis had a corresponding minimal increase in SAA, potentially corresponding to a minor illness. For HP, even when all captive birds were considered clinically normal, all surprisingly had values above the RI calculated. Full medical histories are provided in Supplementary Tables S2–S4, as minor elevations in HP may correlate to subclinical low-grade pododermatitis common in managed captive flocks or to other subclinical disease not diagnosed at the time of sampling. For SAA and HP levels from the same individual, both were higher in clinically abnormal compared to normal states. This suggests possible value in comparing SAA and HP levels over time in individual animals to monitor disease state in captivity or during rehabilitation efforts. An important limitation, however, is that a very small number of rehabilitated animals were assessed here; future studies should aim to investigate the utility of monitoring serum amyloid A and HP among captive flocks and rehabilitated animals, using a larger sample size and variety of disease states.

Serum biochemistry parameters are previously described in seabirds, including Xantus’s murrelets, waved albatrosses (*Phoebastria irrorata*), and a few tropical seabirds (4, 55, 64, 65). Reference intervals in this study were comparable to previous waterbird studies, although amylase was notably higher in the sampled rhinoceros auklets compared to common loons and brown pelicans (4, 58). Scarce information exists regarding amylase concentrations in seabirds, and differences may be attributed to genetic variability within the species and are not necessarily diet-specific (66). Uric acid RI were also higher in rhinoceros auklets than previously reported in common loons, waved albatrosses, dark-rumped petrels (*Pterodroma phaeopygia*), and wedge-tailed shearwaters (*Ardenna pacifica*) (4, 64, 65). Rhinoceros auklets in this study were sampled at night, as they flew back from their burrows after foraging all day. Elevated uric acid could therefore be attributed to postprandial blood sampling as has been observed in captive black-footed penguins (*Spheniscus demersus*) and peregrine falcons (67, 68) or potentially due to dietary differences between those species. Uric acid differences could also reflect variation due to sampling at different times of days. Higher serum triglyceride concentrations in rhinoceros auklets compared to common loons may be reflective of the high lipid content during egg laying and brooding season, as demonstrated in the blue-footed booby (*Sula nebouxii*); alternatively, this may instead, reflect dietary differences between rhinoceros auklets and common loons (4, 69). Subclinical plastic ingestion has also been linked with increased uric acid, amylase, and cholesterol levels in flesh-footed shearwaters (*Ardenna carneipes*) (70). It is unknown whether plastic pollution similarly affects these parameters in rhinoceros auklets, though plastic fibers have been found in the rhinoceros auklet diet (71).

No sex differences in serum biochemical parameters were noted in this study, similar to black-browed albatrosses (*Thalassarche melanophris*) (72). By contrast, sex-related differences have been previously found in waterbirds including higher calcium and triglycerides in adult female Alaskan seabirds and brown pelicans (22, 58), which may potentially indicate species differences or reproductive status differences from rhinoceros auklets.

As IAV is an important pathogen for surveillance in wild birds within the Pacific Flyway, rhinoceros auklets were surveyed for antibodies. We detected seropositivity to IAV antibodies across rhinoceros auklets in all sampling years, indicating viral exposure. This highlights the utility of continued surveillance, especially in light of the H5N1 HPAI outbreak in wild birds across North America and Europe. IAV serology has previously been assessed in free-ranging adult waved albatrosses and southern giant petrels, with no animals testing positive (64, 73). Among different seabird species on the Canadian East coast, Atlantic puffins (*Fratercula arctica*) and common murres (*Uria aalge*) exhibited 22 and 44% antibody prevalence, respectively, while several other species were seronegative (33). It is unclear what the source of IAV exposure is to these rhinoceros auklets. One possibility is that sympatric species such as gulls, known to act as reservoirs for low pathogenic H13 and H16 subtypes of IAV (34), could be a potential source. Additional work is required to understand cross-species transmission and other modes of exposure to IAV.

It is important to note that while many sampled birds had antibodies against IAV, they were all clinically healthy on external examination. Therefore, while it is possible that previous IAV infection could have affected the serum EPH, APP, and biochemical analytes assessed (e.g., increased SAA with acute infection), it is probably less likely unless birds were infected at the time of sampling. Understanding the viral subtypes that rhinoceros auklets are exposed to is particularly important, as it has been experimentally shown in wood ducks that previous exposure to IAV could infer some degree of homosubtypic (homologous hemagglutinin) and heterosubtypic (heterologous hemagglutinin) protective immunity if birds are exposed to H5N1 HPAI (74). Continued serological surveillance is required to understand the role of rhinoceros auklets in IAV epidemiology, and their potential as a reservoir species.

There were several limitations to this study. This study was conducted over multiple years and colonies, and a variety of small inter-colony and inter-annual differences for parameters were observed. Notably, inter-annual differences have also previously been observed in clinical metrics for other seabird species (75, 76). Given the minor variations in these parameters that were not suggestive of biological significance, RI were compiled with inclusion of all individuals to provide a broader representation of rhinoceros auklet populations across multiple years and colonies. There was prolonged storage of some samples prior to testing, which could have affected protein presence and metabolism. However, the impact of sample storage duration is not well described in the literature and therefore was not a variable investigated in this study. Finally, only brief external exams were conducted upon animal capture and so subclinical disease may have been missed. As such, since the EPH RI generated here are broad, there may have been some ill birds within the group sampled. To counteract this limitation in potentially missing animals with subclinical disease, we utilized Tukey’s interquartile fences to identify statistical outliers as per the American Society of Veterinary Clinical Pathology guidelines. Further research is required to compare health parameters between diseased and healthy rhinoceros auklets. For example, the serum EPH, APP, and biochemistry profiles of birds from rehabilitation centers with known injuries should be further characterized to help correlate parameters with clinical disease or injuries. Future research can assess how these health parameters may vary with increased stress in birds, as a proxy for subclinical illness; this can be done by conducting hormone analysis.

In conclusion, this study provides serum EPH, APP, and biochemistry RI for rhinoceros auklets, an important alcid sentinel species of the North Pacific, following recommended guidelines by the American Society of Veterinary Clinical Pathology. Overall, rhinoceros auklet RIs are comparable to other aquatic bird species, although rhinoceros auklets appeared to have higher upper RIs for serum amylase, uric acid, and triglyceride concentrations. This work is critical for wildlife conservation and management in the North Pacific, as it facilitates the monitoring of marine ecosystem health in the face of stressors such as pandemic illness, infectious disease, climate change, plastic ingestion, pollution, increased marine vessel traffic, and large-scale environmental catastrophes such as oil spills. Furthermore, the clinical nature of these data will provide a useful basis for assessment during the rehabilitation of seabirds, especially during climatic or anthropogenic events.

## Data availability statement

The raw data supporting the conclusions of this article will be made available by the authors, without undue reservation.

## Ethics statement

Research protocols employed in this study were approved by Simon Fraser University Animal Care Services (#974B-94), the Western and Northern Animal Care Committee of Environment and Climate Change Canada’s Canadian Wildlife Service (14MH01, and 19MH01), ECCC Migratory Birds banding permit (10667F), and US Fish and Wildlife Federal Bird Banding Permit (22913).

## Author contributions

LL: Data curation, Formal analysis, Investigation, Methodology, Visualization, Writing – original draft, Writing – review & editing. JH: Conceptualization, Formal analysis, Funding acquisition, Investigation, Methodology, Resources, Supervision, Visualization, Writing – original draft, Writing – review & editing. GF: Conceptualization, Funding acquisition, Investigation, Methodology, Writing – original draft, Writing – review & editing. CC: Formal analysis, Methodology, Validation, Writing – original draft, Writing – review & editing. SPe: Data curation, Formal analysis, Supervision, Writing – review & editing. CF: Writing – review & editing. NC: Writing – review & editing. SH: Writing – review & editing. SPa: Formal analysis, Writing – review & editing. DS: Methodology, Writing – review & editing. EF: Writing – review & editing. KH: Conceptualization, Data curation, Funding acquisition, Investigation, Methodology, Resources, Supervision, Visualization, Writing – original draft, Writing – review & editing.
