# Supplementary material for: Baseline health parameters of rhinoceros auklets (Cerorhinca monocerata) using serum protein electrophoresis, acute phase proteins, and biochemistry
Source: Front Vet Sci. 2024 Jun 25;11:1379980. doi: 10.3389/fvets.2024.1379980 (PMC11231077; doi:10.3389/fvets.2024.1379980)
Supplement: Supplementary file 1 [file Data_Sheet_1.docx]

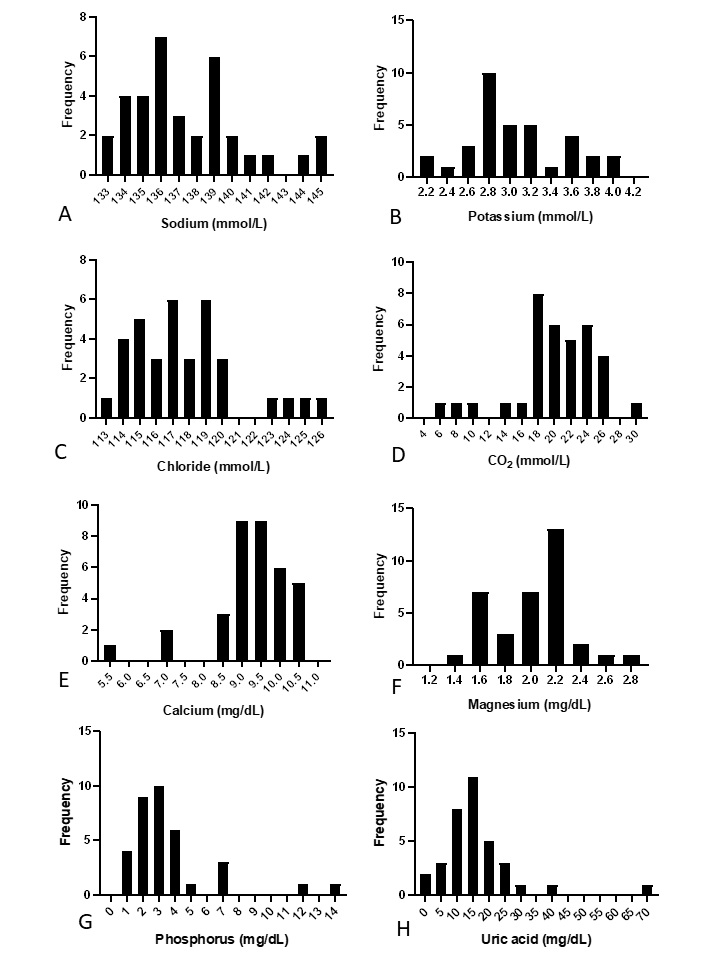


**Figure S1.** Serum biochemistry histograms for all free-ranging adult rhinoceros auklets (*Cerorhinca monocerata*) (*n*=35) captured from Lucy, Pine, Triangle Islands, and SGang Gwaay, British Columbia, Canada from 2013-2019, prior to exclusion of outliers. The number of birds is denoted by frequency on the y-axis. (A) Sodium; (B) potassium; (C) chloride; (D) CO_2_ (one bird was below the limit of detection of 5 mmol/L); (E) calcium; (F) magnesium; (G) phosphorus; and (H) uric acid.


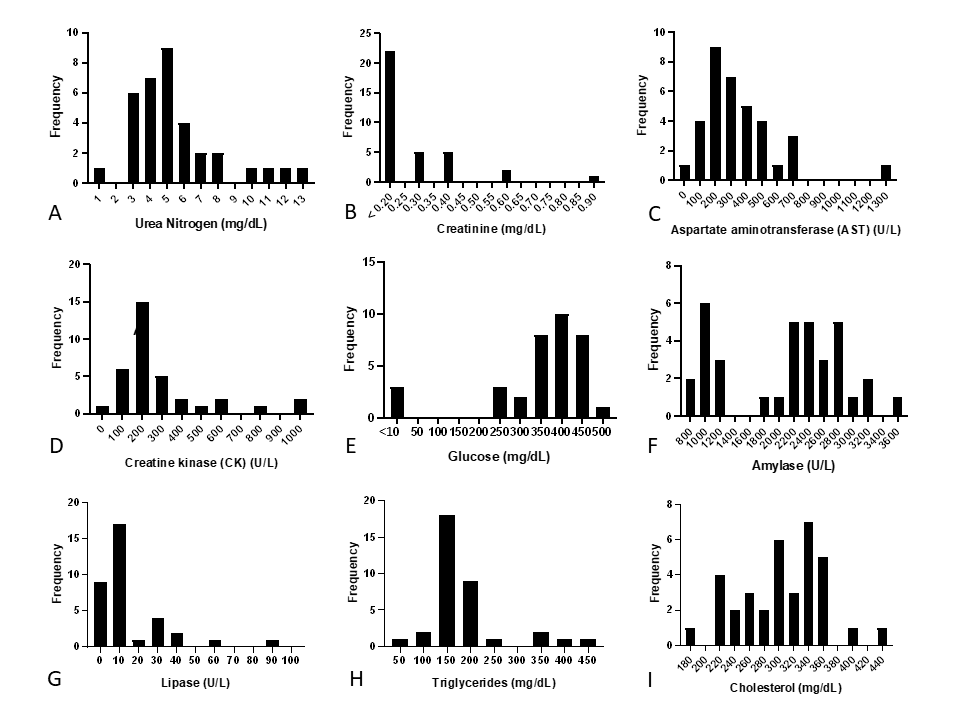


**Figure S2.** Serum biochemistry histograms for all free-ranging adult rhinoceros auklets (*Cerorhinca monocerata*) (*n*=35) captured from Lucy, Pine, Triangle Islands, and SGang Gwaay, British Columbia, Canada from 2013-2019, prior to exclusion of outliers. The number of birds is denoted by frequency on the y-axis. (A) Urea nitrogen; (B) creatinine (13 birds were below the limit of detection of 0.2 mg/dL); (C) aspartate aminotransferase; (D) creatine kinase; (E) glucose (three birds were below the limit of detection of 10 mg/dL); (F) amylase; (G) lipase (two birds were below the limit of detection of 1 U/L); (H) triglycerides; and (I) cholesterol.

**Figure S3.** Serum amyloid A (mg/L) (*n*=156) and haptoglobin (mg/mL) (*n*=148) serum concentrations from all free-ranging rhinoceros auklets (*Cerorhina monocerata*) sampled across Lucy, Pine, Triangle Islands, and SGang Gwaay, British Columbia, Canada, and Protection Island, Washington, USA, including values determined as statistical outliers using Tukey interquartile fences. Birds from Protection Island were not assessed for haptoglobin levels. The limits of detection for serum amyloid A and haptoglobin were <0.1 mg/L and <0.01 mg/mL, respectively.

**Table S1.** Sex determination of wild adult rhinoceros auklets (*Cerorhinca monocerata*) using genetics compared to measurements of bill depth and body mass. Birds with bill depths <16.5 mm and >17.0 mm were predicted to be female and male, respectively. Birds with ambiguous bill depth (16.5-17.0 mm) were predicted to be females if body mass was <500 g.

|  | | **Genetic sex** | | |
| --- | --- | --- | --- | --- |
|  |  | **Female** | **Male** | **Total number of animals** |
| **Sex predicted using morphometric measurements** | **Female** | 12 | 5 | 17 |
|  | **Male** | 5 | 14 | 19 |
|  | **Total number of animals** | 17 | 19 | 36 |

**Table S2.** Signalment and history of five captive rhinoceros auklets (*Cerorhinca monocerata*) from Alaska SeaLife Center, used for preliminary assessment of serum amyloid A and haptoglobin levels before and after resolution of clinical abnormalities.

| Individual ID | Genetics | Sex | Date of birth (mm/dd/yyyy) | Date of death (mm/dd/yyyy) or whether alive as of 03/01/2023 |
| --- | --- | --- | --- | --- |
| CM01 | 1st generation wild - egg collected from Middleton AK for research project, University of Alaska Fairbanks | Male | 6/23/2006 | 6/18/2012 |
| CM02 |  | Male | 6/23/2006 | Alive |
| CM06 |  | Female | 6/27/2006 | 6/16/2016 |
| CM11 |  | Male | 7/6/2006 | Alive |
| CM12 | 2nd generation wild - hatchling of CM06 and CM01 at Alaska SeaLife Center | Female | 7/22/2009 | Alive |

**Table S3.** Serum amyloid A (SAA) and haptoglobin levels in five captive rhinoceros auklets (*Cerorhinca monocerata*) with various clinical abnormalities.

|  | Clinically abnormal captive rhinoceros auklets^a^ | | | | | | |
| --- | --- | --- | --- | --- | --- | --- | --- |
| Individual ID | **Sample date^b^** | **Sample type** | **Age at collection** | **Key clinical concerns at time of sample collection** | **Diagnostic results^b^** | **SAA (mg/L)** | **Haptoglobin (mg/mL)** |
| CM01 | 10/27/2009 | Plasma LiHep | 3 years | *Diagnosis:* Open. Acute weight loss, weeks duration, poor feather grooming.   - Possible exposure to moldy nest material; aspergillosis antibody titer moderate positive but decreased from serial annual testing. Clinical improvement (appetite, preening) seen within first two weeks of 14-day antibiotic and 60-day antifungal therapy. | 08/18/2009:   - CBC: PCV 48%, TP 4.4; tWBC 21,600 (28% H, 68% L, 2% M, 0% E, 2% B). - Aspergillus Panel: No globulinopathies, Aspergillus galactomannan 0.2 (negative), Aspergillus antibody optical density reading of 1.4 (decreased from 2008). - Whole body radiographs: NSF.   10/27/2009:   - Hemolysis index 3+, therefore EPH values not shown | 0.83 | 1.39 |
| CM02 | 06/15/2008 | Plasma LiHep | 2 years | *Diagnosis:* Pododermatitis with active abscessation, *Enterococcus* sp. (2 types) on culture, surgically debrided 05/26/2008 & 11/2008. No radiographic bone involvement.   - Possible exposure to moldy nest material; aspergillosis antibody titer - moderate positive. | 06/15/2008:   - EPH: TP 5.8; albumin to globulin ratio 0.47 g/dL; prealbumin 0 g/dL; albumin 1.86 g/dL; α-1 globulins 0.51 g/dL; α -2 globulins 1.62 g/dL; β-globulins 1.25 g/dL; ɣ-globulins 0.56 g/dL   06/18/2008:   - CBC: PCV 43%, TP 6.0, tWBC 13,086 (62% H, 29% L, 3% M, 0% E, 6% B) - Aspergillus Panel: Aspergillus antibody optical density reading of 1.8 (moderate positive). Aspergillus Galactomannan - 0.2 (negative). | 43.85 | 1.27 |
| CM06 | 08/28/2007 | Serum | 1 year | *Diagnosis:* Cellulitis with swelling of lateral aspect of right foot heel; acute exacerbation of chronic lesion since transfer to facility in 2007.   - Resolved with 14-day antimicrobial therapy. | 08/28/2007:   - CBC: PCV 44%, TP 4.8; heterophilia - tWBC 19,256 (68% H, 29% L, 3% M). - Foot radiographs: Revealed no bony involvement, soft tissue swelling with focal faint suspect mineralization in central aspect of right heel pad. - EPH: TP 2.2 g/dL; albumin to globulin ratio 1.06; prealbumin 0 g/dL; albumin 1.13 g/dL; α -1 globulins 0.10 g/dL; α -2 globulins 0.22 g/dL; β-globulins 0.37 g/dL; ɣ-globulins 0.38 g/dL. | 42.95 | 0.67 |
| CM11 | 05/17/2007 | Plasma LiHep | 1 year | *Diagnosis:* Acute joint infection of TMT-P1 joint, third digit, left foot; *Enterococcus* sp. on culture.   - Acute severe left foot lameness. - Examination: Fluctuant swelling of dorsal aspect of tarsometatarsal-first phalangeal joint of the third digit on the left foot. Persistent, static plantar residual callouses, and chronically thickened heel pad of left foot noted since intake to facility. - Swelling and lameness resolved after 24-day antimicrobial therapy, redirected after culture and susceptibility results reported. | 05/17/2007:   - CBC: PCV 50%, TP 5.8, tWBC 16,720 (81% H, 16% L, 0% M, 0% E, 3% B). - Foot radiographs: Increased left D3 MT-P1 joint space. No bony involvement noted. | 355.07 | 2.45 |
| CM12 | 10/21/2009 | Plasma LiHep | 3 months | *Diagnosis:* Severe pododermatitis with active abscessation with necrosis distal to infection on left foot third digit.   - Surgical debridement on 07/22/2009, noted flexor tendon necrosis at amputation site. Culture growth of *Staphylococcus* sp. (coagulase positive), Mixed flora Gram positive. - On recheck, due to persistent pododermatitis, abscessation, and swelling of the P2-P3 interdigital joint and growth of *Staphylococcus aureus* on joint tap culture, and bone lysis on radiographs despite medical management, a distal toe amputation was performed on 11/18/2009. | 08/18/2009:   - Aspergillus Panel: Aspergillus antibody optical density reading of 0.7 (negative). Aspergillus Galactomannan - 0.1 (negative), Increase in beta globulins (1.12 g/dL) on electrophoresis may indicate underlying acute inflammatory process.   10/21/2009:   - CBC: PCV 38%, TP 7.0, tWBC 16,500 (86% H, 8% L, 1% M, 0% E, 5% B). - Fibrinogen – 300 mg/dL. - Foot radiographs: No bony involvement proximal to abscessation of left foot D3. Soft tissue swelling prominent. - Sample quantity not sufficient for EPH. | 385.13 | 1.6 |

^a^CBC=complete blood count; PCV=packed cell volume; TP=total protein (g/dL); tWBC=total white blood cell count; H=heterophils; L=lymphocytes; M=monocytes; E=eosinophils; B=basophils; NSF=no significant findings; EPH=protein electrophoresis. The Aspergillus Panel with ELISAs was performed at the University of Miami Avian & Wildlife Laboratory.

^b^Dates are listed in order of month, day, and year.

**Table S4.** Serum amyloid A (SAA) and haptoglobin levels in five captive rhinoceros auklets (*Cerorhinca monocerata*) when considered clinically healthy.

|  | Clinically healthy captive rhinoceros auklets^a^ | | | | | | |
| --- | --- | --- | --- | --- | --- | --- | --- |
| Individual ID | **Sample date^b^** | **Sample type** | **Age at collection (years)** | **Clinical notes at time of sample collection** | **Diagnostic results^b^** | **SAA (mg/L)** | **Haptoglobin (mg/mL)** |
| CM01 | 08/20/2008 | Serum | 2 | Annual exam: NSF | 08/20/2008:   - CBC: PCV 44%, TP 4.7, tWBC 7,440 (44% H, 49% L, 3% M, 4% B, 0% E). - Aspergillus antibody optical density of 2.0 (moderate), Aspergillus galactomannan 0.1 (negative), no globulinopathies. | <0.1 | 0.35 |
| CM02 | 09/01/2009 | Plasma LiHep | 3 | Annual exam: Finishing fall molt; blood feathers present for majority of primary and secondaries bilaterally. Persistent, static soft subcutaneous nodule in ventral skin near junction of medial and middle toe. Otherwise, NSF. | 09/01/2009:   - CBC: PCV 40%, TP 5.2, tWBC 14,000 (54% H, 41% L, 0% M, 0% E, 5% B) - Aspergillus Panel: Aspergillus antibody optical density reading of 1.6 (weak positive – decreased compared to serial follow-up from 2008). Galactomannan - 0.2 (negative), alpha 2 globulins on electrophoresis static from 2008 sampling (0.8 g/dl). | 0.49 | 0.87 |
| CM06 | 09/14/2010 | Plasma LiHep | 4 | 09/19/2010: Ophthalmologist examined for right cataract scar originally noted as a quiescent finding in 2008. Diagnosed hazy anterior cortical cataract, likely from historic injury, and not deemed to be significantly interfering with vision. Otherwise, NSF on annual examination. | 2010:   - CBC: PCV 41%, TP 4.1, tWBC 14,080 (38% H, 58% L, 3% M, 0% E, 1% B). | 15.52 | 0.5 |
| CM11 | 08/15/2011 | Plasma LiHep | 5 | 07/25/2011: Annual exam - NSF  08/15/2011: Recheck - NSF. | 07/25/2011:   - CBC: PCV 50%, TP 5.8, tWBC 36,919 (85% H, 8% L, 7% M, 0% E, 0% B). Fibrinogen 400.   08/15/2011:   - Follow-up CBC performed due to increased tWBC noted three weeks prior without any clinical concerns. CBC: PCV 56%, TP 5.4, tWBC 12,358 (46% H, 45% L, 2% M, 0% E, 7% B). | 1.65 | 0.62 |
| CM12 | 09/24/2013 | Plasma LiHep | 4 | 09/24/2013: Annual exam - Recent molt of tail feathers, persistent thickening and scar at distal third digit of the left foot, otherwise NSF. | 09/24/2011:   - CBC: PCV 45%, TP 5.0, tWBC 6242 (86% H, 9% L, 3% M, 2% B). Possible rubricyte or myeloblast cell seen on blood film stained for differential count.   12/31/2013:   - Follow-up CBC performed due to heterophilia. CBC: tWBC 18,409 (37% H, 31% L, 28% M, 0% E, 1% B). | <0.1 | 0.4 |

^a^CBC=complete blood count; PCV=packed cell volume; TP=total protein (g/dL); tWBC=total white blood cell count; H=heterophils; L=lymphocytes; M=monocytes; E=eosinophils; B=basophils; NSF=no significant findings. The Aspergillus Panel with ELISAs was performed at the University of Miami Avian & Wildlife Laboratory.

^b^Dates are listed in order of month, day, and year.
